# Da Vinci robotic assisted pyeloplasty versus laparoscopic pyeloplasty in newborns under 3 months

**DOI:** 10.1038/s41598-025-14603-x

**Published:** 2025-08-04

**Authors:** Boshen Shu, Shufeng Zhang, Jian Gao, Lin Wang, Shuangshuang Wang, Ruoyi Shi, Xiaohui Wang

**Affiliations:** Department of Pediatric Surgery, Henan Provincial People’s Hospital, Zhengzhou, 450003 Henan Province China

**Keywords:** Ureteropelvic junction obstruction, Robot-assisted, Laparoscopy, Newborn, Pyeloplasty, Retrospective study, Urology, Paediatric urology

## Abstract

Ureteropelvic junction obstruction (UPJO) is a common cause of hydronephrosis in children. We aimed to investigate the efficacy of robotic-assisted laparoscopic pyeloplasty (RALP) in newborns with UPJO compared to laparoscopic pyeloplasty (LP). We conducted a retrospective study of newborns aged ≤ 3 months who underwent RALP or LP from May 2018 to December 2023. Only primary pyeloplasty cases were included. Seventy-seven newborns (RALP = 46; LP = 31) were enrolled and no significant difference in the newborns’ demographics and pre-operative parameters was found. The mean operation time (OT) was 161.30 ± 29.07 min (RALP) and 200.60 ± 26.66 min (LP) (P < 0.0001), and the mean hospitalization stay was 7.80 ± 1.13 days (RALP) and 9.32 ± 1.19 days (LP) (P < 0.0001). RALP was associated with a higher hospitalization cost than LP (73449 ± 8513 yuan vs. 40152 ± 7555 yuan; P < 0.0001). The effectiveness and safety of RALP for treating UPJO in newborns is comparable to that of LP. In addition, RALP might have advantages over LP with its faster recovery and less trauma.

## Introduction

 With the development of minimally invasive surgery (MIS), the invention and utilization of the Da Vinci robot-assisted surgical system has marked a new period in surgery^1^. Over the past few years, robot-assisted surgery has been progressively used in children and adults, including urology, arthroplasty, general surgery, and other subgroups^2–4^.

Ureteropelvic junction obstruction (UPJO) is one of the most common causes of hydronephrosis in children^5^. Several meta-analysis studies have indicated that both laparoscopic pyeloplasty (LP) and robot-assisted laparoscopic pyeloplasty (RALP) are feasible options to treat hydronephrosis caused by UPJO in children with the advantages of shorter hospitalization stay and lower morbidity as well as a comparable success rate compared to open pyeloplasty^6,7^. Nevertheless, during LP, it is hard to carry out suturing and surgical knot-tying to finish the anastomosis, which causes a steep learning curve because of a small working place and limited area, particularly in newborns^8^. Due to several advantages such as flexibility, safety, and efficacy compared to LP and OP, RALP is becoming an increasingly popular surgical approach for UPJO which has been broadly accepted^9^. Several previous studies have compared RALP and LP; nonetheless, those studies have concentrated on older children or infants^10,11^. Moreover, to the best of our knowledge, no study has assessed newborns aged 3 months or less specially. In the present study, we aimed to compare the characteristics of RALP and LP in newborns with UPJO.

## Materials and methods

### Patients and clinical data

**Fig. 1:**
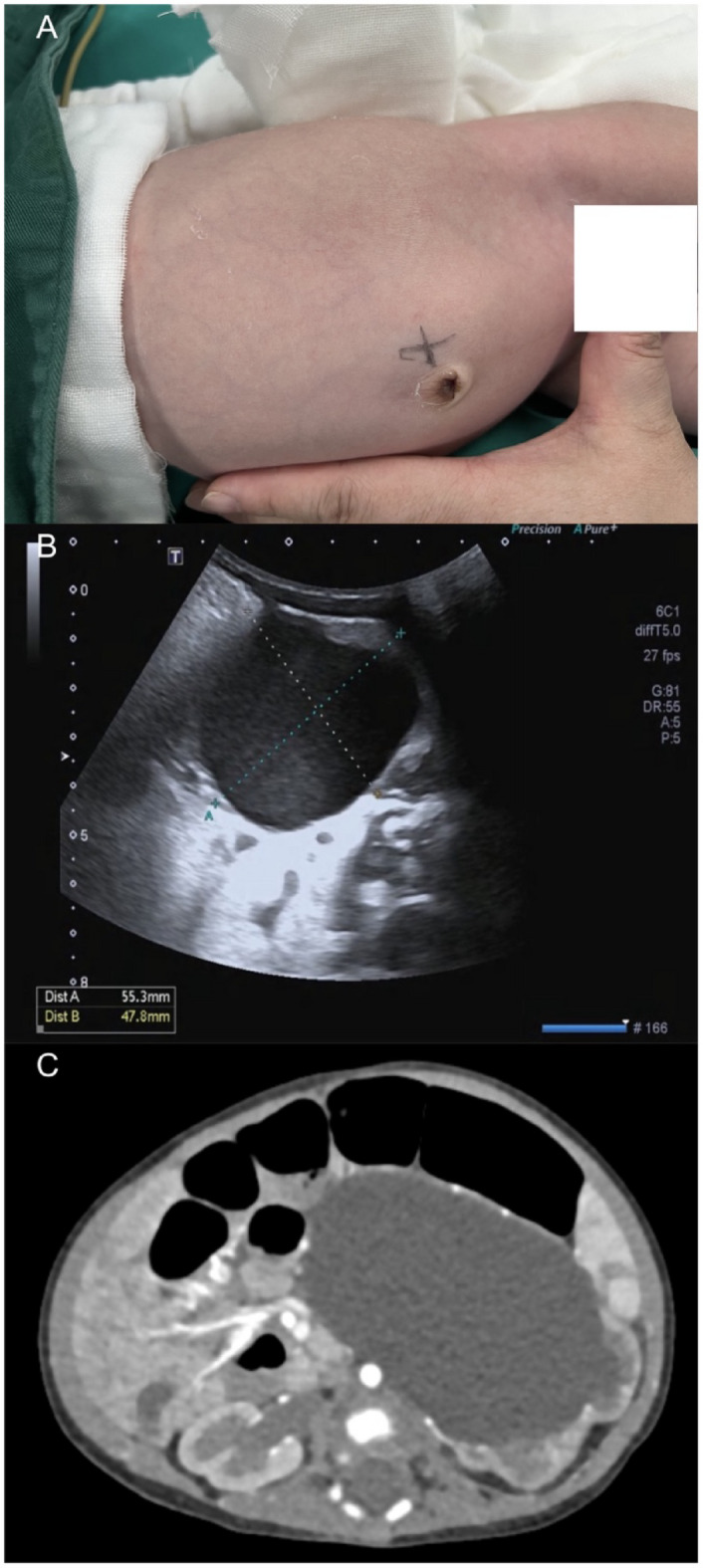
(**A**) The diagnosis presented hydronephrosis (**B**) Preoperative US showed hydronephrosis (**C**) Outcomes of preoperative CT indicated severe hydronephrosis.

Seventy-seven newborns aged 5–90 days who had undergone RALP (*n* = 46) and LP (*n* = 31) from May 2018 to December 2023 in Henan Provincial People’s Hospital were studied retrospectively. Only primary pyeloplasties conducted by or under the supervision of the same senior surgeon, along with similar perioperative management strategies were included in the present study. Ultrasound (US) and CT examination was performed before surgery, the diagnosis presented hydronephrosis caused by UPJO (Fig. 1). Indications for surgery included at least one of the followings: progressive worsening of hydronephrosis in serial US, symptomatic obstruction, split renal function less than 45% and grade III or grade IV hydronephrosis as defined by the Society of Fetal Urology. This study was approved by the Ethics Committee of Henan Provincial People’s Hospital (No.: 2322122014) and was conducted with strict adherence to the tenets of the Declaration of Helsinki. All newborns’ parents agreed informed consents for their data to be used in the present study.

### Surgical technique

**Fig. 2:**
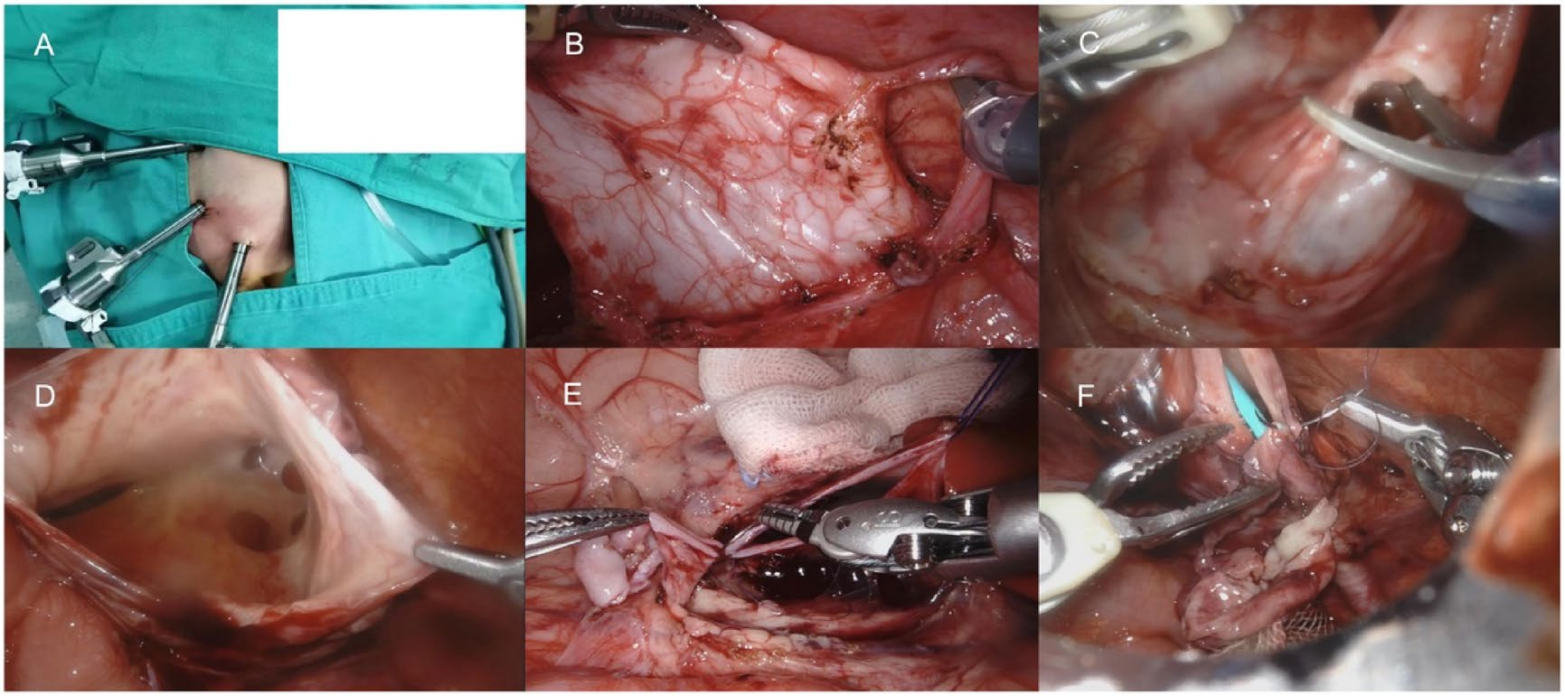
(**A**) Positions of the Da Vinci Trocars (**B**) Identifying the stricture ureter (**C**) Trimming excess renal pelvis (**D**) Exposing the renal pelvis (**E**) Making anastomostomosis of the renal pelvis valve and the lowest point of ureteral cut (**F**) Inserting double J ureteral tubes with the assistance of guidewire.

The newborns were operated under general anesthesia with orotracheal intubation. Surgical steps of RALP and LP were almost the same via transperitoneal approaches. For RALP, after general anesthesia, the child was immobilized in a 30° left-side reclined position with the lumbar region elevated. We inserted 8-mm trocars in the supraumbilical position, inverse McBurney point, and 3 cm below the xiphoid process respectively (Fig. 2). The pneumoperitoneum was established with CO_2_ to 10mmHg, and the 4th generation Xi Da Vinci surgical systems were utilized. Then, dismembered Anderson-Hynes pyeloplasty and double J ureteral tube implantation with the assistance of guidewire were performed, followed by placing a pararenal pelvis drainage tube (Fig. 2). The operation time (OT) was defined as the time interval from the initial incision to the completion of skin suture. For LP, a 5-mm trocar was used for camera access, and two more 3-mm instrument ports were placed at sub-xiphoid and suprainguinal region. A transmesenteric approach was taken to expose the renal pelvis and distinguish the UPJO. The double J ureteral tube (size: F4.7 × 14) was routinely placed before the completion of laparoscopic pyeloplasty.

### Prognosis and follow-up

After surgery, routine anti-inflammatory management was conducted, and the pararenal pelvis drainage tube was removed when the drainage volume was no more than 15 mL for two continuous days. Patients were discharged several days later after pararenal pelvis drainage tubes were removed. The OT, blood loss, perioperative complications, postoperative hospitalization stay, and hospitalization cost were recorded for each patient. The double J ureteral tube was removed under local anesthesia and cystoscopic control according to the patient’s situation between 4 and 6 weeks after surgery. Besides, all patients were evaluated by urinalysis and US at postoperative 1 month, 3 months, and 6 months routinely. Surgery was marked successful when symptoms were resolved, hydronephrosis was reduced or resolved, improved drainage, and stable or decreasing APD.

### Statistical analysis

Continuous numerical data is expressed either as median alongside its range or as mean with standard deviation (±). All characteristics of RALP and LP were compared via Student’s t-test or the Mann–Whitney U test as appropriate. Linear regression analysis was applied to study the correlation between OT and increasing case experience in RALP or LP. P values of < 0.05 were considered as statistically significant.

## Results

**Fig. 3:**
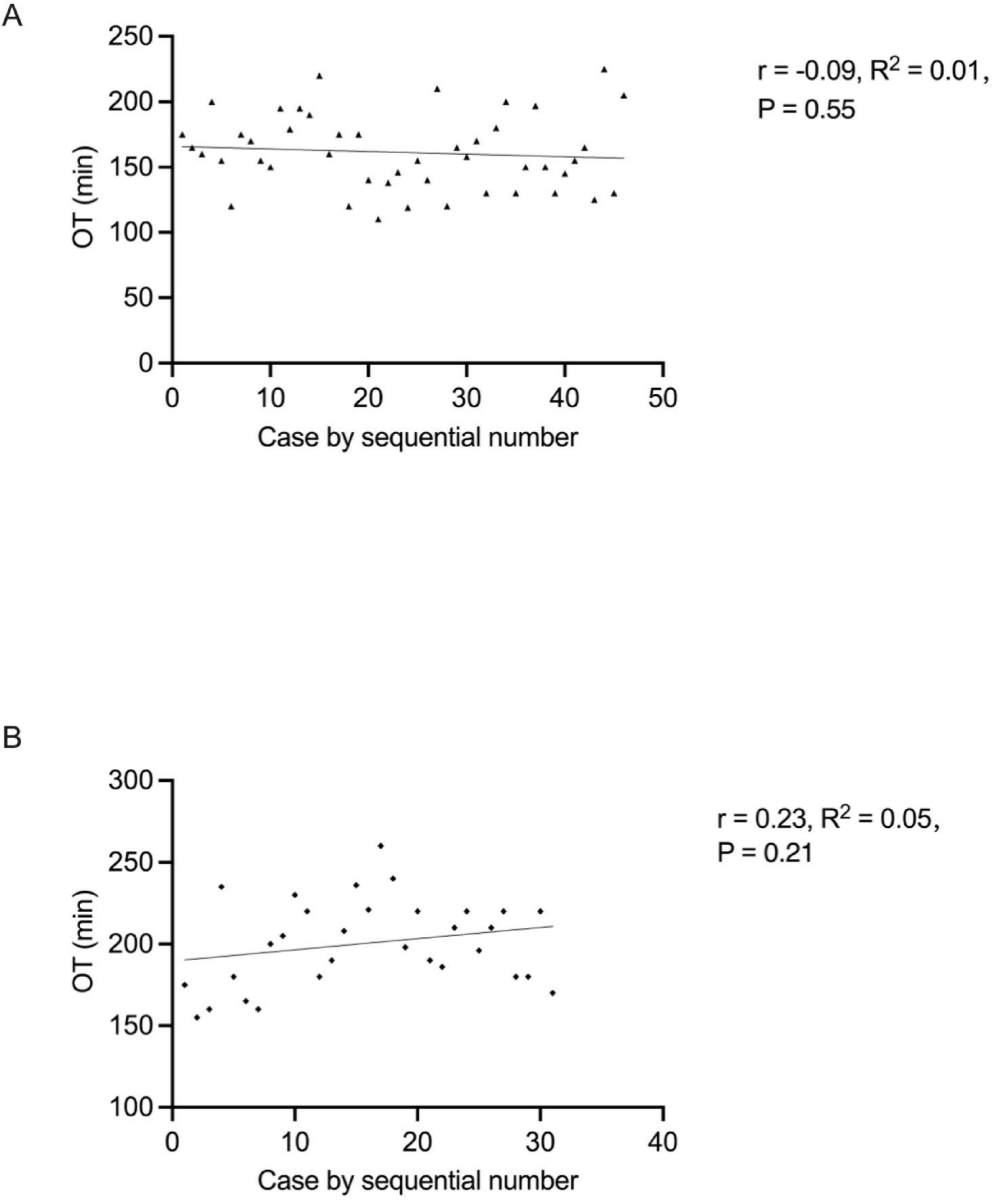
There were no significant correlations between OT and increasing case experience of RALP (*P* = 0.55) (A) or LP (*P* = 0.21) (B).

A total of 77 newborns (RALP = 46; LP = 31) were included in the present study with medians of age and body weight at 46 days (5–90 days) and 5.0 kg (3.0–7.5 kg), respectively. No significant difference in the newborns’ demographics and pre-operative parameters was found between these two groups (Table 1). All newborns underwent RALP or LP successfully without conversion to OP and only two cases in LP group had complications (fever) during postoperative period. The perioperative parameters and post-pyeloplasty outcomes were shown as Table 2. The mean OT was 161.30 ± 29.07 min (RALP) and 200.60 ± 26.66 min (LP) (*P* < 0.0001), and the average hospitalization stay was 7.80 ± 1.13 days (RALP) and 9.32 ± 1.19 days (LP) (*P* < 0.0001). In the RALP group, the mean time until removal of the pararenal pelvis drainage tube was 3.09 ± 0.96 days. while it was 4.29 ± 1.16 days for the LP group (*P* < 0.0001). RALP was associated with a higher hospitalization cost than LP. The mean blood loss was 4.52 ± 2.23 mL (RALP) and 10.71 ± 1.77 mL (LP) (*P* < 0.0001). There were no significant correlations between OT and increasing case experience of RALP (*P* = 0.55) or LP (*P* = 0.21) from the linear regression analysis (Fig. 3). The APD has improved significantly at 3 and 6 months after surgery in both groups while the post-operative analgesic requirement presented no significant difference. The success rates of RALP and LP group were 100% and 93.5%, respectively.

**Table 1:** Summary of the pre-operative characteristics and demographics between the two groups.

	RALP group *n* = 46	LP group *n* = 31	*P*-value
Age at the time of surgery (days) (range)	43.11 (5–90)	47.94 (20–90)	0.33
Body weight at the time of surgery (kg) (range)	5.01 (3.0-7.5)	4.91 (3.8–6.4)	0.66
Gender: male/female	42/4	24/7	0.09
Laterality: left/right	33/13	24/7	0.58
Pre-operative APD (mm) (mean ± SD)	29.12 ± 13.19	26.28 ± 5.81	0.26

RALP: robot-assisted laparoscopic pyeloplasty; LP: laparoscopic pyeloplasty; APD: anteroposterior diameter; SD: standard deviation.

**Table 2:** Summary of the perioperative parameters and post-pyeloplasty outcomes between the two groups.

	RALP group *n* = 46	LP group *n* = 31	*P*-value
OT (minutes) (mean ± SD)	161.30 ± 29.07	200.60 ± 26.66	< 0.0001
Tube drainage stay (days) (mean ± SD)	3.09 ± 0.96	4.29 ± 1.16	< 0.0001
Hospitalization (days) (mean ± SD)	7.80 ± 1.13	9.32 ± 1.19	< 0.0001
Blood loss (mL) (mean ± SD)	4.52 ± 2.23	10.71 ± 1.77	< 0.0001
Hospitalization cost (CNY) (mean ± SD)	73,449 ± 8503	40,152 ± 7555	< 0.0001
Postoperative APD (mm) (mean ± SD)	12.40 ± 7.03	12.20 ± 4.70	0.89

RALP: robot-assisted laparoscopic pyeloplasty; LP: laparoscopic pyeloplasty; APD: anteroposterior diameter; OT: operation time; SD: standard deviation.

## Discussion

The first dismembered pyeloplasty was described by Anderson-Hynes in 1949^12^. From that time, it has become the most applied method and the golden standard procedure for the therapy of UPJO. The first LP case applied in children was described by Peters et al. in 1995, with the benefits of less pain and trauma, more rapid recovery time, along with better cosmetic outcomes than those of open surgery^13^. Based on these advantages, laparoscopy has been widely used in numerous urological surgeries. Nevertheless, due to a small working place and limited area, laparoscopy has a steep learning curve and higher technical requirements for surgeons, especially when it is used in children^8^. Gettman et al. firstly reported the application of RALP in children in 2002^14^. Previous studies have proven that RALP could reduce the learning curve for surgeons owning to the flexibility and stability of the robot arm equipment as well as the superiority of ergonomics^15,16^. Consequently, RALP has progressively become a popular choice for the therapy of UPJO and has been accepted by surgeons in pediatric urology field globally^10,17^. However, to our knowledge up to now, the existing studies about RALP in pediatrics are mostly derived from comparative studies with OP, and comparative studies with older children^11,18^. Our study is the first single-institution retrospective study to compare RALP versus LP in newborns aged 3 months or less. Additionally, we enrolled patients managed by the same surgeon to lessen the confounding bias attributed to variations in surgical procedures and post-operative managements that occurred in cross-institutional studies.

In our retrospective study, we found the success rate of LP was 93.5%, which was lower than the average success rate of LP reported by former research in children^19^. The success rate of LP was worried whether it could be lower in infants or newborns than in older children^20^. One of the possible reasons is there were very few studies focused on newborns, the success rates of LP in newborns may have been overestimated. Moreover, the global decreasing interest and a challenging learning curve in LP has inhibiting effects for LP research in children^21^. Only two cases in the LP group had postoperative complications (fever) and as a common complication in minimally invasive surgery, this may be due to the digestive tract system of infants is not well developed, and if abdominal distension occurs for a long time after surgery, it is easy to cause intestinal flora imbalance and endotoxin absorption, resulting in fever^22,23^. For the infants with relatively low body weight, there are several anesthesia challenges such as hypothermia, hyperoxia and hypoglycemia. Hence, intraoperative monitoring of blood glucose, prewarming equipment including overhead radiant warmer, warming mattress and fluid warmers, as well as adjusting inspired oxygen concentration should be applied to avoid these complications^24^. The mean OT was 161.30 ± 29.07 min and 200.60 ± 26.66 min in the RALP group and LP group (the statistical difference was significant). The mean OT of the RALP group was even shorter after excluding the robot installation time. Several previous studies have also indicated the mean OT in the RALP group has advantages over LP group^11,25^. For LP, the laparoscopic intraabdominal suturing was more challenging due to the restricted operative area in young children, which may cause longer OT and greater risks in newborns. There was no significant trend of decrease in OT with increasing experience in RALP or LP group according to the linear regression analysis in our study, which was different from previous report^10^. This may be explained by several potential hypotheses, including a propensity towards increased mental fatigue, decreased compliance with evidence-based medicine and decreased stress tolerance^26^. However, we believe the mean OT will decrease with more surgical cases when our sample size is big enough in the future.

In our study, the mean time until removal of the pararenal pelvis drainage tube was significantly shorter in the RALP group (3.09 ± 0.96 days) than for the LP group (4.29 ± 1.16 days) (*P* < 0.0001). For the average hospitalization stay, there was a statistically significant but slight difference between RALP and LP group (7.80 vs. 9.32 days). It is necessary to explain this slight difference with caution. Given the small size number of each group, any outliers probably have considerable impact to the statistical analysis. Even though our study only included cases operated by or under the supervision of the same surgeon, the possibility of a minor change in discharge criteria over time needed to be considered. However, based on our subjective experience, RALP improves the accuracy in tissue approximation and suturing, and therefore has the potential to make patients recover in a shorter time by reducing tissue damage. Notably, the postoperative hospital stay after the removal of the drainage tube was relatively long in our study. The present findings suggest that preterm status and low birth weight may be independent risk factors for prolonged hospital stay for infants, as evidenced by the well-established correlation between these two factors and poor clinical outcomes^27,28^. Correspondingly, the extended duration of hospital stay contributes to an escalation in the overall cost of hospitalization.

Using 5- or 8-mm trocars in infant RALP remains controversial^10^. A smaller incision is considered as an outstanding advantage for applying 5-mm trocars, however, a longer intracorporeal length for articulation is needed owing to its pulley system and decreased intra-abdominal working space should also be discussed, which is the concern raised by several clinicians^29,30^. We have not utilized the 5-mm trocars which are not supported by the current robotic surgical platform. No significant difference was found for the post-operative analgesic requirement related to the use of 8-mm trocars in RALP when compared with LP using 5- and 3-mm instruments in our study. Nonetheless, it is still of vital importance to develop micro robotic instruments specific for newborns and small children.

RALP also has several disadvantages, including high costs and size of robotic instruments^31^. RALP was associated with a higher average hospitalization cost than LP in our study. The higher hospitalization cost results in a heavy burden on the patient’s family, and the introduction of expensive robotic systems makes its broad application difficult. Additionally, the Da Vinci system we used has no force feedback; thus, the surgeon cannot feel sense of touch, which might cause tissue harm and line breaks during operation. However, with developments of science and technology, robotic surgical systems will continuously be improved so they are less invasive and more convenient; hence, they will probably become hotspots in MIS area.

There were several limitations in the present study including the nature of retrospective study design, small sample size over a long review period, and some, but most likely insignificant dissimilarity in follow-up procedures. Besides, since we only used data from a single institution, some of the potential bias cannot completely be ruled out although we applied the standardized surgical techniques and management protocols.

## Conclusion

To the best of our knowledge, this is the first retrospective study that evaluated the effectiveness of RALP and LP in newborns with UPJO aged 3 months or less particularly. The effectiveness and safety of RALP is comparable to that of LP, and RALP may own several advantages over LP with its faster recovery as well as less trauma. In summary, RALP is expected to become the trend for MIS in the future.

## Data Availability

The datasets generated and/or analysed during the current study are not publicly available due to the policy from our institution but are available from the corresponding author on reasonable request.

## Abbreviations

APD: Anteroposterior diameter
LP: Laparoscopic pyeloplasty
MIS: Minimally invasive surgery
OT: Operation time
RALP: Robotic-assisted laparoscopic pyeloplasty
UPJO: Ureteropelvic junction obstruction
US: Ultrasound

## References

[1] Shu, B., Feng, X., Martynov, I., Lacher, M. & Mayer, S. Pediatric minimally invasive Surgery—A bibliometric study on 30 years of research activity. *Children* **9**, 1264 (2022). 36010154 10.3390/children9081264 PMC9406539

[2] Shu, Q. Robotic-assisted surgery in pediatrics: current applications, limitations and prospects. In *Pediatric Robotic Surgery 1–5* (Springer Nature Singapore, 2023). 10.1007/978-981-19-9693-1_1.

[3] Esposito, C. et al. Robotics and future technical developments in pediatric urology. *Semin Pediatr. Surg.* **30**, 151082 (2021). 34412879 10.1016/j.sempedsurg.2021.151082

[4] Shu, B., Ou, X., Shi, S. & Hu, L. From past to digital time: bibliometric perspective of worldwide research productivity on robotic and computer-assisted arthroplasty. *Digit Health* **10**, 1–11, (2024). 10.1177/20552076241288736 PMC11456188 39372812

[5] Costigan, C. S. & Rosenblum, N. D. Understanding ureteropelvic junction obstruction: how Far have we come? *Frontiers Urology* **3**, 1154740 (2023). 10.3389/fruro.2023.1154740 PMC12327334 40778044

[6] Sun, M. et al. The efficacy of robotic-assisted laparoscopic pyeloplasty for pediatric ureteropelvic junction obstruction: a systematic review and meta-analysis. *Pediatr. Surg. Int.* **39**, 265 (2023). 37673951 10.1007/s00383-023-05541-8

[7] Huang, Y., Wu, Y., Shan, W., Zeng, L. & Huang, L. An updated meta-analysis of laparoscopic versus open pyeloplasty for ureteropelvic junction obstruction in children. *Int. J. Clin. Exp. Med.* **8**, 4922–4931 (2015). 26131065 PMC4483847

[8] Turner, R. M., Fox, J. A. & Ost, M. C. Advances in the surgical pediatric urologic armamentarium. *Pediatr. Clin. North. Am.* **59**, 927–941 (2012). 22857839 10.1016/j.pcl.2012.05.011

[9] Zhou, L., Huang, J., Xie, H. & Chen, F. The learning curve of robot-assisted laparoscopic pyeloplasty in children. *J. Robot Surg.* **18**, 97 (2024). 38413450 10.1007/s11701-024-01856-3

[10] Wong, Y. S., Pang, K. K. Y. & Tam, Y. H. Comparing Robot-Assisted laparoscopic pyeloplasty vs. laparoscopic pyeloplasty in infants aged 12 months or less. *Front Pediatr* **9**, 647139 (2021). 10.3389/fped.2021.647139 PMC8236621 34195160

[11] Sun, L. et al. Laparoscopic versus robot-assisted pyeloplasty in infants and young children. *Asian J. Surg.* **46**, 868–873 (2023). 36192267 10.1016/j.asjsur.2022.09.046

[12] Anderson, J. C. & Hynes, W. RETROCAVAL URETER:A case diagnosed pre-operatively and treated successfully by a plastic operation. *Br. J. Urol.* **21**, 209–214 (1949). 18148283 10.1111/j.1464-410x.1949.tb10773.x

[13] Peters, C. A., Schlussel, R. N. & Retik, A. B. Pediatric laparoscopic dismembered pyeloplasty. *J. Urol.* **153**, 1962–1965 (1995). 7752371

[14] Gettman, M. T., Neururer, R., Bartsch, G. & Peschel, R. Anderson-Hynes dismembered pyeloplasty performed using the Da Vinci robotic system. *Urology* **60**, 509–513 (2002). 12350499 10.1016/s0090-4295(02)01761-2

[15] Chahal, B. et al. The learning curves of major laparoscopic and robotic procedures in urology: a systematic review. *Int. J. Surg.* **109**, 2037–2057 (2023). 37132184 10.1097/JS9.0000000000000345 PMC10389344

[16] Tobias-Machado, M., Mitre, A. I., Rubinstein, M., Costa, E. F. & Hidaka, A. K. da Robotic-assisted radical prostatectomy learning curve for experienced laparoscopic surgeons: does it really exist? *Int Braz J Urol* 42, 83–9 (2016). 10.1590/S1677-5538.IBJU.2014.0485 PMC4811230 27136471

[17] Langreen, S. et al. Laparoscopic pyeloplasty in neonates and infants is safe and efficient. *Front Pediatr* **12**, 1397614 (2024). 10.3389/fped.2024.1397614 PMC11310035 39132308

[18] Bansal, D. et al. Infant robotic pyeloplasty: comparison with an open cohort. *J. Pediatr. Urol.* **10**, 380–385 (2014). 24268880 10.1016/j.jpurol.2013.10.016

[19] Andolfi, C., Adamic, B., Oommen, J. & Gundeti, M. S. Robot-assisted laparoscopic pyeloplasty in infants and children: is it superior to conventional laparoscopy? *World J. Urol.* **38**, 1827–1833 (2020). 31506749 10.1007/s00345-019-02943-z

[20] He, Y. et al. Primary laparoscopic pyeloplasty in children: A single-center experience of 279 patients and analysis of possible factors affecting complications. *J. Pediatr. Urol.* **16**, 331e1–331e11 (2020). 10.1016/j.jpurol.2020.03.028 32334969

[21] Bindi, E. et al. Has robot-assisted pyeloplasty reached outcome parity with laparoscopic pyeloplasty in children <15 kg? A Paediatric YAU international multi-center study. *J Pediatr Urol* 20, 1154–1159 (2024). 10.1016/j.jpurol.2024.09.008 39307658

[22] Mantica, G., Ambrosini, F., Parodi, S., Tappero, S. & Terrone, C. Comparison of safety, efficacy and outcomes of robot assisted laparoscopic pyeloplasty vs conventional laparoscopy. *Res. Rep. Urol.* **12**, 555–562 (2020). 33204662 10.2147/RRU.S238823 PMC7667144

[23] Pawar, D. Common post-operative complications in children. *Indian J. Anaesth.* **56**, 496 (2012). 23293390 10.4103/0019-5049.103970 PMC3531006

[24] Kinouchi, K. Anaesthetic considerations for the management of very low and extremely low birth weight infants. *Best Pract. Res. Clin. Anaesthesiol.* **18**, 273–290 (2004). 15171504 10.1016/j.bpa.2003.12.010

[25] Silay, M. S., Danacioglu, O., Ozel, K., Karaman, M. I. & Caskurlu, T. Laparoscopy versus robotic-assisted pyeloplasty in children: preliminary results of a pilot prospective randomized controlled trial. *World J. Urol.* **38**, 1841–1848 (2020). 31435732 10.1007/s00345-019-02910-8

[26] Maruthappu, M. et al. The influence of volume and experience on individual surgical performance. *Ann. Surg.* **261**, 642–647 (2015). 25072442 10.1097/SLA.0000000000000852

[27] Cotten, M. Prolonged hospital stay for extremely premature infants: risk factors, center differences, and the impact of mortality on selecting a Best-Performing center. *J. Perinatol.* **25**, 650–655 (2005). 16079906 10.1038/sj.jp.7211369

[28] Bhatti, K. M. et al. Factors responsible for the prolonged stay of surgical neonates in intensive care units. *Sultan Qaboos Univ. Med. J.* **15**, e91–e97 (2015). 25685393 PMC4318614

[29] Kawal, T. et al. Robotic surgery in infants and children: an argument for smaller and fewer incisions. *World J. Urol.* **38**, 1835–1840 (2020). 31016450 10.1007/s00345-019-02765-z

[30] Boysen, W. R. & Gundeti, M. S. Robot-assisted laparoscopic pyeloplasty in the pediatric population: a review of technique, outcomes, complications, and special considerations in infants. *Pediatr. Surg. Int.* **33**, 925–935 (2017). 28365863 10.1007/s00383-017-4082-7

[31] Esposito, C. et al. Robot-assisted vs laparoscopic pyeloplasty in children with uretero-pelvic junction obstruction (UPJO): technical considerations and results. *J. Pediatr. Urol.* **15**, 667e1–667e8 (2019). 10.1016/j.jpurol.2019.09.018 31734119

